# Development and validation of the Medical Home Care Coordination Survey for assessing care coordination in the primary care setting from the patient and provider perspectives

**DOI:** 10.1186/s12913-015-0893-1

**Published:** 2015-06-07

**Authors:** Ianita Zlateva, Daren Anderson, Emil Coman, Khushbu Khatri, Terrence Tian, Judith Fifield

**Affiliations:** Weitzman Institute, Community Health Center, Inc., Middletown, CT USA; Ethel Donaghue TRIPP Center, University of Connecticut Health Center, Farmington, CT USA

**Keywords:** Care coordination, Survey research and design, Primary care, Psychometrics, Program Evaluation, PCMH, Safety net/Federally Qualified Health Centers

## Abstract

**Background:**

Community health centers are increasingly embracing the Patient Centered Medical Home (PCMH) model to improve quality, access to care, and patient experience while reducing healthcare costs. Care coordination (CC) is an important element of the PCMH model, but implementation and measurability of CC remains a problem within the outpatient setting. Assessing CC is an integral component of quality monitoring in health care systems. This study developed and validated the Medical Home Care Coordination Survey (MHCCS), to fill the gap in assessing CC in primary care from the perspectives of patients and their primary healthcare teams.

**Methods:**

We conducted a review of relevant literature and existing care coordination instruments identified by bibliographic search and contact with experts. After identifying all care coordination domains that could be assessed by primary healthcare team members and patients, we developed a conceptual model. Potentially appropriate items from existing published CC measures, along with newly developed items, were matched to each domain for inclusion. A modified Delphi approach was used to establish content validity. Primary survey data was collected from 232 patients with care transition and/or complex chronic illness needs from the Community Health Center, Inc. and from 164 staff members from 12 community health centers across the country via mail, phone and online survey. The MHCCS was validated for internal consistency, reliability, discriminant and convergent validity. This study was conducted at the Community Health Center, Inc. from January 15, 2012 to July 15, 2014.

**Results:**

The 13-item MHCCS - Patient and the 32-item MHCCS - Healthcare Team were developed and validated. Exploratory Structural Equation Modeling was used to test the hypothesized domain structure. Four CC domains were confirmed from the patient group and eight were confirmed from the primary healthcare team group. All domains had high reliability (Cronbach’s α scores were above 0.8).

**Conclusions:**

Patients experience the ultimate output of care coordination services, but primary healthcare staff members are best primed to perceive many of the structural elements of care coordination. The proactive measurement and monitoring of the core domains from both perspectives provides a richer body of information for the continuous improvement of care coordination services. The MHCCS shows promise as a valid and reliable assessment of these CC efforts.

**Electronic supplementary material:**

The online version of this article (doi:10.1186/s12913-015-0893-1) contains supplementary material, which is available to authorized users.

## Background

The Patient Centered Medical Home (PCMH) model has been widely implemented to guide system-wide primary care redesign [1] because of its emphasis on team-based care, “whole person” orientation, access, self-management, and coordination of care in a complex health system. While evidence is still relatively limited, some studies of the PCMH model have demonstrated improvements in health care quality [2, 3], access to care [4], patient and staff experience [5, 6], and health care expenditures [2, 3, 7–9]. Studies on PCMH implementation suggest the potential for system-wide benefits, but there are many challenges and barriers that primary care practices must overcome to successfully adopt this model.

Care coordination (CC), one of the core elements of the PCMH model, is particularly challenging in the current healthcare system due to the disjointed, uncoordinated nature of care between multiple providers including primary care, specialists, hospitals, emergency rooms, pharmacies and others. As an example, a recent study found that a typical primary care provider (PCP) shares and coordinates patient care with 229 other physicians [10]. In addition, there is an inadequate exchange of patient information between PCPs and specialists [11]. Given these difficulties, it is unsurprising that few practices standardize care coordination processes for patients. Only about 3 % of small-to-medium-sized primary care practices use care managers [12], and 46 % of larger practices coordinate care for patients with chronic illnesses [13]. For safety-net community health centers, which often service the sickest patients, the challenge of coordinating care is further compounded by the psychosocial and financial issues more commonly faced by these patients. Medically underserved patients are more likely to live and cope with poverty, inadequate housing, unemployment, limited access to specialty care, and linguistic and cultural barriers [14]. These factors contribute to the general poor health that characterizes these patients and place added burden on providers seeking to coordinate and manage their care.

Implementing a CC process in primary care is further complicated by multiple models with different conceptual emphases and a surfeit of evaluation frameworks [15]. To address the dearth of clarity, the Agency for Healthcare Research and Quality (AHRQ) published the *Care Coordination Measures Atlas* [16]*.* In this report, the authors review theoretical frameworks that underscore the study of CC, develop a standardized definition and outline a broad range of measures for evaluating various domains of CC. However, none of these tools fully address the assessment needs of primary care practices looking to implement and monitor CC programs. Furthermore, while there are 22 surveys cited within this report that relate specifically to the Medical Home, the authors call for further study to help determine the applicability of such measures. Building on and complementing the *Atlas,* a systematic review of existing CC measures identified 96 different instruments, the majority of which rely on survey data (88 %) and are applicable to primary care settings (58 %) [17]. Some of these measures were further included in the 2014 update of the *Atlas* [18]*.* However, none of these tools provide a comprehensive assessment of all relevant CC domains in the primary care setting or from the perspective of health care professionals.

To address these problems, we sought to develop and validate a new measurement survey to assess the core domains of CC for primary care practices involved in Medical Home transformation. This new survey, the Medical Home Care Coordination Survey (MHCCS), assesses the perspective of the healthcare team (MHCCS-H) and the patient’s perspective (MHCCS-P). Since many elements of CC relate to activities best perceived by the healthcare team, and since the ultimate output of a CC program is experienced by the patients themselves, the authors believe that both perspectives are important and needed to comprehensively evaluate the coordination of care.

## Methods

**Fig. 1:**
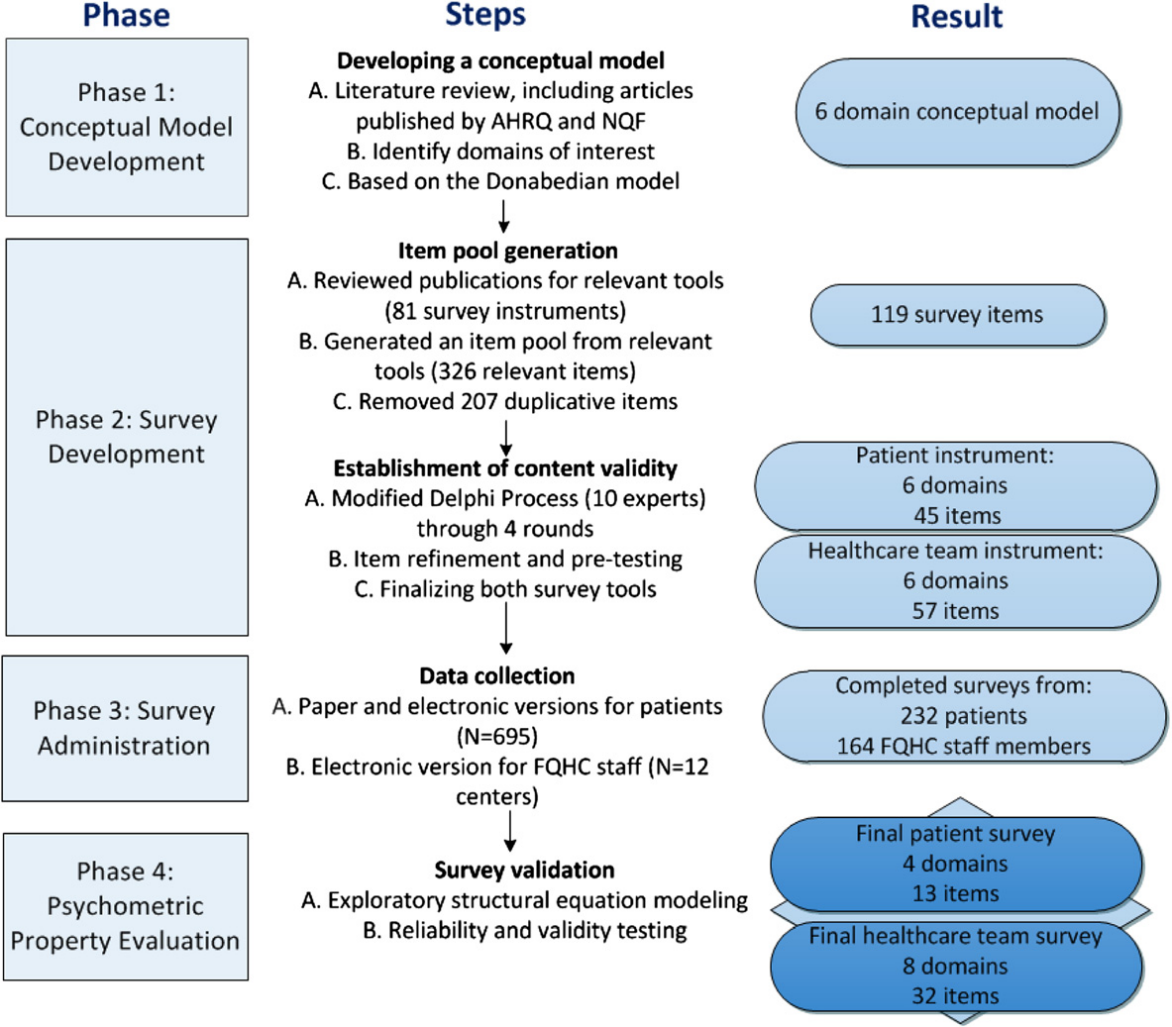
Schematic of the project methods and select results

We conducted this study in several steps: 1) development of a conceptual model; 2) generation of the item pool; 3) review of the items and establishment of content validity; 4) administration of the survey; and 5) psychometric structural evaluation. Figure 1 outlines select methodological steps taken in this project.

### Development of conceptual model

Care coordination is a blanket term that encompasses a wide range of elements that may be assessed. To help frame our work and inform the process of developing and validating CC measures for the primary care safety-net setting, we created a PCMH CC Conceptual Model (Additional file 1). This model uses the consensus AHRQ definition of CC, which is “the deliberate organization of patient care activities between two or more participants (including the patient) involved in a patient’s care to facilitate the appropriate delivery of health care services. Organizing care involves the marshaling of personnel and other resources that are needed to carry out all required patient care activities and is often managed by the exchange of information among participants responsible for different aspects of care” [16]. In developing this model, we conducted an extensive literature review and built on concepts from the *Care Coordination Measures Atlas* [16] and the National Quality Forum (NQF) [19].

We utilized the NQF-endorsed framework for CC that establishes five domains essential to measurement: healthcare home, the use of a proactive plan and follow-up of care, communication between all members of the healthcare team and the patients, care transitions, and information systems [19]. In addition, we based our conceptual model on the dominant theoretical model in health services research, the Donabedian model [20], which emphasizes a systems-level perspective on the determinants of healthcare quality. According to this model, care management structures combined with defined care management processes produce desired outcomes. We adapted the Donabedian Model by identifying essential CC structures (inputs) and process factors (activities) with the potential to affect patient and staff satisfaction as well as clinical and financial outcomes.

**Fig. 2:**
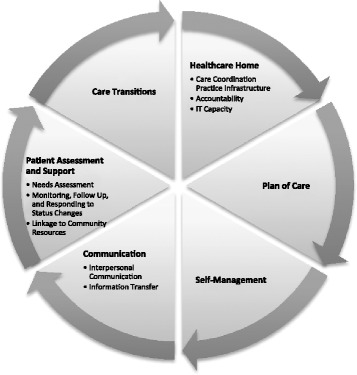
Depiction of the PCMH Care Coordination Conceptual Model

The following domains were selected for inclusion in the measures: (1) Healthcare Home; (2) Plan of Care; (3) Self-Management; (4) Communication; (5) Patient Assessment and Support; and (6) Care Transitions. Three of the domains were further divided into subdomains. The Healthcare Home domain was divided into CC Practice Infrastructure, Accountability, and IT Capacity subdomains. The Communication domain consisted of the Interpersonal Communication and Information Transfer subdomains. The Patient Assessment and Support domain included the Needs Assessment, Linkage to Community Resources, and Monitoring, Follow Up, and Responding to Status Change subdomains (see Fig. 2). After identifying all domains that could appropriately be assessed by primary healthcare team members and their patients, we described the structures (inputs) and processes (activities) involved in those domains and subdomains, and the possible short-term and long-term outcomes (see Additional file 1).

### Generation of the item pool

The newly created PCMH CC Conceptual Model was used as a foundation to design the survey, with the goal of ensuring each element of the conceptual model was appropriately reflected in the new survey from the perspectives of the patient, the administrative staff, and the clinical personnel. Two researchers independently reviewed existing survey instruments from the *Atlas* [16] and from an updated literature search. From these tools, they selected potentially appropriate items for inclusion. The *Atla*s review included 3448 articles, from which 78 potentially useful survey instruments were identified. The literature search strategy described in the *Atlas* [16] was used to update the review and identified 861 additional articles (for the period from January 2010 to May 2012), from which three additional potentially useful survey instruments were selected. Appropriate permission was obtained to include and/or modify items from the instruments that were selected. From the selected 81 survey instruments, we identified 326 potentially useful items. After removing redundant items, 119 items remained for possible inclusion. Each item was independently mapped by each of the two reviewers to one domain in the conceptual model, with a consensus process used when differences were observed. New items were developed where important constructs lacked specific measures and to ensure that each domain and subdomain in the model contained at least two items. Questions were also reworded so that they had a consistent structure for the Delphi process.

### Review of the items and establishment of content validity

In order to examine content validity we adopted an anonymous, web-based Delphi Technique, which is an iterative method to help derive consensus in areas that lack sufficient scientific evidence [21–23]. We used a modified electronic version of the Delphi technique to obtain expert opinion and consensus regarding the design of the final survey. To recruit participants for the Delphi process, we identified experts in the field of CC and PCMH based on their having significant publications, a national/international profile, and/or substantial clinical/practical experience in the field. Of the 16 invited experts, three declined participation, three did not respond, and ten agreed to participate.

To carry out each of the four rounds of the Delphi process we used REDCap (Research Electronic Data Capture) [24], a secure, web-based application designed to support data capture for research studies. In the first three rounds, experts reviewed the pool of items and rated, on a 5-point Likert scale, each item’s appropriateness and ability to assess the indicated element of the conceptual model. Participants could also suggest that an item be reworded, moved to another domain, or eliminated. After each round, items that received an “Appropriate” or “Very Appropriate” rating from 80 % or more of the experts were accepted for inclusion in the measures, while items that received an “Inappropriate” or “Very Inappropriate” rating from more than 50 % of the experts were removed. The items that did not reach consensus either way were presented again to the experts for review in the next round. After each round, questions were modified and reworded based on the qualitative input from the experts. In the final round, experts commented on the general format, language, response options, skip patterns and definitions used.

The Delphi participants confirmed the hypothesized domain-subdomain structure for the MHCCS. The MHCCS - P comprised 45 questions over six distinct domains, while the MHCCS - H comprised 57 items over six domains. The major difference between the two models was the absence of the “Information Technology Capacity” subdomain in the patient version.

Finally, cognitive interviews were conducted with three patients who had received CC services at the Community Health Center, Inc. to verify the ease of comprehension of the survey items, survey instructions, study information sheet, definition of key terms, skip patterns and response options. Their input led to a final version of the survey instrument. All survey questions were worded to be written at a fourth grade reading level.

In both versions of the MHCCS, all care coordination questions were structured in a 5-point Likert scale format. The last few questions of each survey were multiple-choice and represented demographic and control variable information. These selected questionnaire items were not included in the analyses undertaken to identify the final domain structures, and instead provided support for the convergent and predictive validities.

### Administration of the survey

Patients were recruited from a large, multi-site Federally Qualified Health Center (FQHC) located in Connecticut. Community Health Center, Inc. (CHCI) provides comprehensive primary care services in 12 health centers across the state and over 200 additional sites including school-based clinics, homeless shelters, and mobile outreach sites. CHCI cares for over 130,000 medically underserved patients in the state. Over 60 % of CHCI patients are racial/ethnic minorities; over 90 % are below 200 % of the federal poverty level, 60 % are on Medicaid or state insurance, and 22 % are uninsured. This study was reviewed and approved by the Institutional Review Board at CHCI and conducted from January 15, 2012 to July 15, 2014.

Inclusion criteria for patients were: age 18 years or older, having English as a preferred language and a medical visit at CHCI within the past 12 months. In addition to these criteria, patients had to have had at least one of the following: 1) two or more emergency room visits in the past year; 2) a hospitalization in the past year; 3) diabetes with a hemoglobin A1C test result greater than 9 % in the past 6 months; 4) four or more of the following chronic illnesses: chronic obstructive pulmonary disease (COPD), hypertension, asthma, diabetes and coronary artery disease (CAD). A total of 695 eligible patients were randomly chosen through the electronic health record system and were invited to complete the survey either on paper or online. Patients were incentivized to complete the MHCCS-P, as they would be entered into a raffle to win one of five $50 gift cards. Patients who did not return the survey two weeks after the initial mailing were sent a second survey, followed by a reminder phone call. Patients who failed to complete the survey after this round received a second reminder survey in the mail, followed by a second phone reminder. During this final reminder call, patients were also offered the opportunity to complete the survey over the phone. In total, 232 surveys were completed for a response rate of 33.4 %. The responders’ socio-demographic and inclusion characteristics are reported in Table 1.

**Table 1:** Patient responder characteristics

		Total (%)
		(N = 232)
Gender	Female	133 (57)
	Male	99 (43)
Ethnicity	Black or African American	44 (19)
	Caucasian	141 (61)
	Hispanic	38 (16)
	Other	5 (2)
	Unreported	4 (2)
Age	20-29	6 (3)
	30-39	13 (6)
	40-49	48 (21)
	50-59	89 (38)
	60-69	51 (22)
	70+	25 (11)
Educational Level	No schooling	3 (1)
	Grade 1 to 12	53 (23)
	High school or GED completed	72 (31)
	Some college	51 (22)
	Associate’s degree	15 (6)
	Bachelor’s degree	14 (1)
	Advanced degree	2 (1)
	Other	14 (6)
	Missing	8 (3)
Inclusion Categories^a^	Hospitalized	122 (53)
	2 or more ER Visits	69 (30)
	A1C above 9	46 (20)
	4 Chronic Illnesses^†^	22 (9)

^a^ Some patients fit two or more inclusion criteria

^†^ Four or more of the following chronic illnesses: chronic obstructive pulmonary disease (COPD), hypertension, asthma, diabetes and coronary artery disease (CAD)

The healthcare team survey (MHCCS –H) was administered at CHCI and 11 other large, multi-site FQHCs across the country. At each site, the Chief Executive Officer (CEO) or the Chief Medical Officer (CMO) was asked to email the invitation to complete the online survey to all PCPs, nurses, and clinical administrative staff. After approximately five weeks, the lead contact person at each site was asked to email a reminder to all staff to complete the survey. Respondents’ characteristics are summarized in Table 2.

**Table 2:** Healthcare team responder characteristics

		Total (%)
		(N = 164)
Gender	Male	27 (16)
	Female	97 (59)
	Unknown	40 (24)
Self-Identified Roles	Administrator	15 (9)
	Nurse (e.g., RN,LPN)	21 (13)
	Nurse Care Coordinator	6 (4)
	Nurse Practitioner (e.g., APRN)	16 (10)
	Physician Assistant	8 (5)
	Primary Care Physician (e.g., MD, DO)	40 (24)
	Other	16 (10)
	Unknown	42 (26)

Of the 162 invited CHCI staff members, 55 returned completed surveys for a 34 % response rate. An additional 109 completed surveys were received from the other 11 health centers invited to participate. These health centers were unable to provide the total number of staff who were offered the survey, preventing calculations of a final response rate.

Respondents to the MHCCS indicated their consent to participate in the study by completing the survey.

In addition, clinical and operational data were obtained from CHCI’s EHR and practice management system.

### Psychometric and structural validation

Data were analyzed using IBM SPSS Statistics 20 Software Package [25] and Mplus 7.11 [26]. An initial sense of how items clustered in domains was provided by comparing correlations of each item with items from the same hypothesized domain to correlations with items from other domains, or the within-domain vs. between-domains average correlations. The quantitative validation of the measures followed current psychometric standards [27, 28], grounded in classical test theory [29, 30], which involve primarily testing the reliability and validity of the measure. Since the very structures of the measures were meant to be validated (i.e., which items cluster under what specific domain), classic reliability indices like Cronbach’s α alone would be inadequate for measures that are not first proven to be unidimensional. The structure of multi-dimensional measures (and hence their unidimensionality) is best tested in the Structural Equation Modeling (SEM) framework [31–33]. The SEM approach assumes the existence of unobserved (latent) variables that explain the correlations between a group of effect indicators, i.e., the observed items assumed to belong to the same domain [34]. A range of exploratory and confirmatory factor analyses (EFA and CFA) can be tested on the data to estimate both the number of domains (or factors) and what questions belong to what factor or confirm a specific number of domains, with a more or less specified structure per factor known a priori. We employed such a sequence of models, starting with a full CFA model to test the structure as initially hypothesized, then moved into a more advanced recent combination of the two, called Exploratory Structural Equation Modeling (ESEM) [35]. ESEM can group the survey items (indicators) into a pre-specified number of factors (in our case, the hypothesized domains), while allowing items to belong simultaneously to different domains, and incorporating correlations between item residuals. We tested ESEMs with increasingly more domains for both the patient and healthcare team CC domains, until a clear structure emerged, based on large enough standardized loadings and explained variance (or R^2^), which led to final CFA models. The reliability of the domains that emerged from the final models was then assessed. Convergent validity of the CC domains was tested by confirming sizeable correlations with other measures that the domains were expected to correlate with; specifically for the MHCCS-P, whether patients have a care plan, a rating of the level of CC received (agreement, on a 1-5 scale), and a question rating the care received (from poor = 1 to excellent = 5), and for MHCCS-H, a rating of the care coordination provided (from poor = 1 to excellent = 5), one question asking whether someone in the practice coordinates care (agreement, on a 1-5 scale), and the role of the staff in the practice. Discriminant validity of the CC domains was tested by determining that no relationships existed with unrelated concepts; specifically for MHCCS-P, patients’ gender and ethnicity, and for MHCCS-H the gender of staff. Lastly, predictive validity or the ability to predict other relevant outcomes was tested for MHCCS-P only, with a health rating item (from poor = 1 to excellent = 5).

## Results

### Survey validation

Cronbach’s α of the proposed domains are measures of internal consistency, as they reflect average inter-item correlations. Pure confirmatory factor analyses of the hypothesized structures of the patient and healthcare team CC measures were rejected, so a sequence of ESEM models were tested (syntax and output are available at trippcenter.uchc.edu/modeling). When loadings were non-significant (less than .5 in standardized values), and R^2^ were small (less than .5, or 50 % of the variance in that item explained by the latent factor), items were dropped from that factor. CFA models were iteratively trimmed by dropping items that were not explained well by the model and regrouping items when less than three items were left in a factor/domain.

Starting with 45 questionnaire items in the MHCCS-P, 32 were dropped from the reliability and internal consistency testing. For the MHCCS-H, these numbers were 57 and 25, respectively. Thus, the final MHCCS-P consisted of 13 items, and the MHCCS-H consisted of 32 items.

The final results are shown in Tables 3 and 4. The MHCCS-P and MHCCS-H can be found in Additional files 2 and 3 in their final format. Four distinct patient CC domains and eight provider CC domains emerged from the final analyses, with the following four being common between CC measure types: Plan of Care (PC), Communication (Comm), Link to Community Resources (ComRes), and Care Transitions (CT). The provider CC measure had four additional domains: Accountability (Acc), IT capacity (IT), Follow-up PC (FPC), and Self-Management (SM); a total CC score was computed for each CC measure type as the average of all domains. Internal consistency was high for all MHCCS-P domains (Cronbach alphas = .893 - .909) and for all MHCCS-H domains (Cronbach alphas *= .803 - .903).* All four final domains and the global CC score correlated (from .377 to .708, p < .001) with whether patients had a care plan, and with the ratings of the care received and of the care coordination received. Overall, CC domains and CC scores did not differ by patient education level or ethnicity, with the exception of the Communication domain, supporting MHCCS-P’s discriminant validity. The predictive validity of the MHCCS–P was also confirmed because the global CC score and three of the four domains, all except Care Transitions (r = .048, *p* = .653), correlated with the self-rated health (.235, *p* < .001 with PC; .272, *p* < .001 with Comm; .177, *p* < .001 with ComRes).

**Table 3:** Structure of the final four domain patient survey as emerged from analyses

	Care coordination domain	Items	λ	R^2^
1	Plan of Care (PC) *α = .909*	My PCT (Primary Care Team) helps me plan so I can take care of my health	.93	.87
		My PCT follows through with the care plan it creates with me	.89	.78
		Someone on my PCT helps me set goals for taking care of my health	.92	.85
		My PCT asks for my ideas when we make a plan for my care	.88	.78
2	Communication (Comm) *α = .899*	Someone on my PCT tells me all my test results, good and bad	.97	.94
		I get the results of my lab tests in a timely manner	.95	.89
		Someone on my PCT helps me understand what my lab tests	.70	.50
3	Link to Community Resources (ComRes) *α = .893*	Someone on my PCT gives me information about services offered at their office or in my community	.89	.80
		Someone on my PCT asks me about what I need for support	.87	.76
		Someone on my PCT encourages me to attend programs in my community	.78	.60
4	Care Transitions (CT) *α = .893*	After I leave the hospital, my PCT knows about new prescriptions or if there was a change	.94	.89
		After I leave the hospital, my PCT helps me get back on my feet	.85	.73
		After I leave the hospital, my PCT knows about the care I received from the hospital	.66	.43

All four domains correlated pairwise with each other significantly (*p* < .001) and moderately (.44 to .75); the *χ* ^2^ (54) = 66.7, *p* = .115, CFI = .987, RMSEA = .046, 95 % CI [.001; .078], five pairs of indicators’ errors were correlated

**Table 4:** Structure of the final 8 domain Healthcare Team survey as emerged from analyses

	Domain	Items	λ	R^2^
1	Accountability (Acc) *α = .844*	The PCT (Primary Care Team) team is made up of members with clearly defined roles, such as patient self-management, education, proactive follow up and resource coordination.	.72	.52
		The PCT and patients share responsibilities in managing patients’ health.	.74	.55
		The PCT is characterized by collaboration and trust.	.78	.60
		The PCT works with patients to help them understand their roles and responsibilities in care.	.74	.55
2	IT capacity (IT) *α = .874*	The PCT uses electronic data to monitor and track patient health indicators and outcomes.	.83	.69
		The PCT team uses electronic data to support the documentation of patient needs. ^N^	.75	.56
		The PCT uses electronic data to develop care plans. ^N^	.79	.63
		The PCT uses electronic data to determine clinical outcomes. ^N^	.90	.80
3	Plan of Care (PC) *α = .903*	The PCP asks for patients’ input when making a plan for their care. ^N^	.82	.67
		The PCT helps make care plans that patients can follow in their daily life. ^N^	.89	.80
		The PCT develops care plans that incorporate plans recommended by other health care providers patients see. ^N^	.91	.83
4	Follow-up Plan of Care (FPC) *α = .886*	The PCT team reviews and updates patients’ care plan with them. ^N^	.81	.65
		The PCT follows through with the care plan. ^N^	.74	.55
		The PCT uses patients’ care plan to follow progress. ^N^	.80	.64
		The PCT helps patients plan so they can take care of their health even when things change or when unexpected things happen.	.78	.62
5	Self-Management (SM) *α = .803*	Someone on the PCT team helps patients set goals for managing their health.	.77	.60
		Someone on the PCT team checks to see if patients are reaching their goals.	.75	.56
		The primary care practice/health center has behavior change interventions readily available for patients as part of routine care.	.61	.37
		The primary care practice/health center has peer support readily available for patients as part of routine care.	.68	.46
6	Communication (Comm) α = .865	The PCT team informs patients about any diagnosis in a way that patients can understand.	.78	.61
		The PCT team helps patients understand all of the choices for their care.	.78	.61
		*The PCT team considers and respects patients’ values, beliefs and traditions when recommending treatments.*	*.74*	*.55*
		*The PCT team’s care coordination activities are based upon ongoing assessment of patient needs.*	*.75*	*.56*
7	Link to Community Resources (ComRes) *α = .896*	*Someone on the PCT team offers patients the opportunity to learn more about managing their health, such as with group appointments, support groups and patient education.*	*.74*	*.54*
		Someone on the PCT team asks patients about what they need for support, such as care programs, financial services, equipment and transportation.	.79	.62
		Someone on the PCT team gives patients information about additional supportive services offered at the practice/health center or in their community, such as counseling programs, support groups or rehabilitation programs.	.86	.75
		Someone on the PCT team encourages patients to attend programs in their community that could help them, such as support groups or exercise classes.	.79	.63
		Someone on the PCT team connects patients to needed services, such as transportation or home care.	.83	.69
8	Care Transitions (CT) α = .875	When patients are discharged from the hospital, the PCT team is informed about the care patients received from the hospital.	.69	.48
		When patients are discharged from the hospital, the PCT team receives information from the hospital about new prescriptions or if there was a change in medication.	.68	.47
		When patients are discharged from the hospital, their primary care medical record includes a discharge summary in a timely manner. ^N^	.91	.83
		When patients are discharged from the hospital and there are test results pending, their primary care medical record includes the test results within 2 weeks. ^N^	.85	.72

Indicators in italics were originally hypothesized to belong to a different domain; ^N^: items had never/always response options, while the others had the disagree/agree options; fit was *χ* ^2^ (417) = 639.3, *p* < .001, CFI = .931, RMSEA = .058, 95%CI [.049; .067]; 19 pairs of residual errors were correlated

The MHCCS-H domains (and the overall CC score) showed good convergent validity, all being correlated with the rating of the care coordination provided (from .402 to .628, all *p v*alues < .001), and with how the practice coordinates care (from .433 to .630, all *p* values < .001). Moreover, there were differences by staff role in Accountability (Acc), IT capacity (IT), Follow-up Plan of Care (FPC), Link to Community Resources (ComRes), and the overall provider CC score (CC), with average scores ordered as follows: Administrator (highest) > Nurse (middle) > PCP (lowest) for all domains and the overall CC score. No differences were seen by gender of respondent, except for females reporting higher healthcare team CC average scores for the Plan of Care (PC), Follow-up PC (FPC), and Communication (Comm) domains, confirming discriminant validity. The predictive validity of MHCCS-H needs further investigation.

## Discussion

We developed the MHCCS-P and MHCCS-H for assessing the provision of CC in the primary care safety-net setting from the perspectives of patients and the healthcare team, and examined each survey’s construct validity among the patient sample at a large FQHC and among a clinical staff sample from 12 FQHCs across the country. The resulting models provided a reasonable fit and revealed satisfactory levels of internal consistency reliability. The self-report survey provides a framework for evaluating the coordination of care for patient populations requiring complex care within the primary care setting and in critical transitions. The MHCCS-P and MHCCS-H are, to our knowledge, the first to incorporate a broad range of CC domains and provide a comprehensive, non-condition-specific assessment for primary care. They hold the potential to be of particular use to primary care practices seeking a practical tool to help assess CC in the medical home environment.

In this study, we used a consensus approach to select appropriate CC domains and develop measures specific to the outpatient community health setting, and then validated these measures in a real-world practice environment. At the time this study was conducted, CHCI was implementing a standard CC model as part of its adoption of the PCMH model. It thus provided an ideal testing environment for the new measures.

Although some domains were collapsed or discarded during the ESEM analyses, most were maintained in the final version of the healthcare team survey. Similarly, key domains that suitably represent some of the most salient features of the PCMH CC Conceptual Model such as Care Plan, Communication, Patient Assessment and Support and Care Transitions, were retained in the final version of the patient survey. The ESEM analyses, however, excluded from the patient survey two domains that can be essential to the model from a clinical and a PCMH perspective – the Self-Management and Healthcare Home domains. This may suggest that these individual domains may be less perceived by patients, or less visible to them as standalone domains of care as measured by the survey items. We tried to analytically re-attach items from the discarded domains to the final patient survey structure, as it was strongly suggested during the survey development process that they were clinically relevant and conceptually consistent. The resulting models were rejected purely on statistical grounds, because the items’ removal helped to clarify the structure of the domains that were retained. We recognize that the poor performance of the rejected items may have been due to the nature of the study sample rather than the properties of the items. While we think practitioners and researchers should continue to validate the structure of the MHCCS-P as emerged from our analyses with other samples of patients, we also suggest they consider alternative solutions by including some of the items that were rejected with the current model.

Healthcare reform efforts are shifting the emphasis to accountable care. This shift, combined with incentives to implement the PCMH model and obtain recognition from agencies such as the National Center for Quality Assurance (NCQA) are leading to a growing interest in improving care coordination across the healthcare continuum. As primary care practices seek to implement CC within the PCMH model of care, increased attention and support will be needed to assist them with implementation of key features, including a well-functioning team that focuses on the patient’s needs while using evidence-based practices. Strategies will be needed to enable teams to function effectively in this mode and help them establish accountability and negotiate responsibilities for the desired outcomes with their patients. Actively engaging patients and their families as members of the medical home care team and the medical neighborhood is critical to the PCMH model. Similar to the CAHPS Patient-Centered Medical Home Survey (PCMH CAHPS) [36] the MHCCS-H asks about care provided by the entire primary care team, not just the primary care clinician. Items loading on the Accountability domain in the healthcare team survey reflect the fact that care coordination involves multiple different members of the care team, including the patient. The Accountability items pertain to collaboration and working together in new ways, and gauge healthcare team members’ ability to successfully share responsibilities in managing patients’ health as a team.

Patient-centered outcomes are critical for a more balanced assessment of healthcare quality [37]. Since CC is essentially dedicated to identifying patient needs and helping to meet those needs on an individual case-by-case basis, patient feedback should be an essential part of any evaluation. Our MHCCS-P incorporates such patient feedback in assessing the quality of CC. We recruited patients in the study exclusively from one large multi-site FQHC and achieved a response rate of 33.4 %, which was close to the rates (35-44 %) reported for low-income populations in the Consumer Assessment of Health Plans Study (CAHPS) [38]. We used a similar low-income, low-literacy patient population to validate the survey. Such patients have higher rates of chronic illness, poorer health outcomes overall [14], and are more likely to require support in the form of CC than patients in the general population. While this is one of the strengths of this study, it is also a limitation in that the results may not be generalizable to wider patient populations. In addition, it is important to note that the MHCCS-P was validated with data collected in one region of the country and that the characteristics of participating patients may have differed from those of patients who chose not to participate. Performance of this survey, including rejected MHCCS-P items, should be reevaluated in a more diverse primary care patient population.

While the use of risk screening tools is a promising method [39], there is no one best method for identifying patients in need of CC within the medical home. Individual patients may have a need for different forms of CC (either simultaneously or at different points in time). A patient recently discharged from the hospital may need brief transition care support, while a patient with poorly controlled chronic illness may need disease management, self-management support and a care plan as well as links to community resources and supports. These complex needs led us to select a comprehensive set of patient inclusion criteria, which in turn allowed for variation in the level of received CC (patients received low level, intermediate and high-level care coordination).

To better capture the process of CC and its quality, practices should consider using the MHCCS-P and MHCCS-H together. Assessments of CC processes that are more difficult to capture in a survey can benefit from advanced analytical approaches to yield additional insight into contextual factors that facilitate or impede CC. Combining MHCCS data with quantitative data may provide the most thorough and balanced assessment of CC quality in primary care. The exact measures and data collection methods need to be determined based on the purpose for the CC assessment [40].

A major strength of the MHCCS-P and MHCCS-H is that they assess all relevant domains of CC. Given that the individual domain measures performed well, individual components of the survey may be able to be used on their own.

It is particularly worth noting that concurrently with this work, the NQF released a revised CC framework and priorities for CC measurement in September 2014 [41]. Their final conceptual framework includes eight subdomains: Comprehensive Assessment, Goal-Setting, Shared Accountability, Linkages/Synchronization, Quality of Services, Experience, Progression toward Goals and Efficiency. Each of these subdomains maps onto a corresponding domain or subdomain in the PCMH CC Conceptual Model used in this project. Additionally, the multi-stakeholder CC reviewing committee recommended deliberate action to fill performance gaps in addressing four of these eight domains: Comprehensive Assessment, Shared Accountability, Linkages/Synchronization, and Progression toward Goals. The final domain structure of the MHCCS-P upholds and addresses each of these: Plan of Care, Accountability, Link to Community Resources and Follow-up Plan of Care, respectively.

Further research is needed to assess whether the individual domain scores and total CC scores improve in response to a CC intervention, whether survey scores are associated with clinical outcomes, satisfaction in care, and healthcare costs and savings, and to explore the feasibility of a single dyadic patient-provider CC measure [42–44].

## Conclusions

In conclusion, we developed the MHCCS-P and MHCCS-H, with questions mapped to each domain of a broad conceptual model of CC. Our findings suggest that the framework has both clinical as well as construct validity. The MHCCS-P and MHCCS-H were designed to measure quality of CC from the perspectives of the patients and the healthcare team. Both instruments demonstrated good reliability and discriminant validity in this first field test. They can be used separately or together to evaluate CC strengths and areas for improvement within the medical home practice. Although developed and validated for measuring CC at FQHCs, the survey instruments may be relevant for measuring CC among other primary care populations. Further studies are needed to determine whether the survey can detect clinically important changes over time.

## Additional files

Additional file 1: **PCMH Care Coordination Conceptual Model.**

Additional file 2: **Final MHCCS-P.**

Additional file 3: **Final MHCCS-H.**
