# Supplementary material for: Development and validation of the Medical Home Care Coordination Survey for assessing care coordination in the primary care setting from the patient and provider perspectives
Source: BMC Health Serv Res. 2015 Jun 7;15:226. doi: 10.1186/s12913-015-0893-1 (PMC4482098; doi:10.1186/s12913-015-0893-1)
Supplement: Supplementary file 1 — PCMH Care Coordination Conceptual Model. [file 12913_2015_893_MOESM1_ESM.pdf]

Additional file 1. PCMH Care Coordination Conceptual Model

PCMH Care Coordination Conceptual Model

Care Coordination Domains		Structure (Inputs)	Process (Activities)	Short Term Outcomes	Long Term Outcomes
**Healthcare Home**	Care Coordination Practice Infrastructure	- has the function of care coordination embedded into its organizational structure either on site or remotely<br>- has a procedure to identify high risk patients who need care coordination services, based on clinical outcomes, utilization and/or cost<br>- has documented policies and guidelines to direct care coordination activities<br>- provides access to designated care coordinators to support high risk patients<br>- has primary care teams that collaborate to bring coordinated care to patients<br>- has a designated function to assist patients with referrals to specialists<br>- has a tracking system to track and monitor critical referrals	- identifies patients needing care coordination<br>-Care coordinators work closely with primary care providers to coordinate and manage care for complex patients needing additional support<br>-High risk patients receive care coordination support from a designated care coordinator<br>-Care coordinators assist patients with needed referrals<br>-Care coordinators track and monitor critical referrals	-Patients at high risk for adverse outcomes are identified and receive frequency contact and support<br>-Improved patient satisfaction	-Improved clinical outcomes<br>-Reduced hospital/ER utilization<br>-Reduced costs
	Accountability	- has clearly defined roles and responsibilities for patient care (e.g. provision of chronic and preventive care, supporting self-management, coordinating care)	- negotiates roles and responsibilities of each member of the primary care team, including the patient<br>-Primary care team, including the patient, understands roles and responsibilities that each member of the team has for patient care and carrying out the care plan	-Improved staff satisfaction<br>-Improved communication and trust	-Improved patient outcomes (e.g. chronic disease outcomes, preventive care measures, hospital/ER utilization rates)

Care Coordination Domains		Structure (Inputs)	Process (Activities)	Short Term Outcomes	Long Term Outcomes
	IT Capacity	- has a computerized information system (e.g. electronic medical records or registry) to identify patients needing care coordination, track their outcomes, document patient education and self-management, and record plans of care	-Patients receive care from a primary care team that uses technology and information systems to assist in meeting their needs	-Clinical decisions are supported by data<br><br>-Medical records are accessed in a timely fashion<br><br>-Care plans are better documented<br><br>-Information transfers are timely and accurate<br><br>-Increased staff satisfaction	-Reduced costs and hospital/ER utilization<br><br>-Improved clinical outcomes, quality, safety, and efficiency of care<br><br>-Reduced medical errors<br><br>-Improved care delivery processes<br><br>-Reduced duplicative testing
**Plan of Care**		- has a documented care plan for each patient receiving care coordination that is available to all members of the primary care team, including the patient<br>- provides access to care coordinators who establish, review and update care plans for all patients receiving care coordination services	-Care coordinators meet with appropriate care team members, including patient and PCP, to establish a care plan<br><br>-Care coordinators review and update care plans on a recurring basis<br><br>-Patients receive a copy of their care plan	-Care plans are better documented<br><br>-Communication amongst all participants in patient's care is enhanced<br><br>-Improved patient satisfaction	-Improved chronic disease outcomes<br><br>-Reduced hospital/ER utilization<br><br>-Increased medication adherence
**Self-Management**		- has a process to provide patient self-management education and support for all appropriate patients (e.g. peer mentoring and counseling groups)<br>- provides access to care coordinators or other patient educators to instruct patients on self-management	-Care coordinators and/or other primary care team members educate and empower patients with chronic illnesses to take an active role in managing their illness through self-management education, problem-solving skill development, and goal setting<br><br>-Patients with chronic illness meet face to face with care coordinators or other patient educators for self-management education<br><br>-Patients work with the primary care team to	-Enhanced patient self-efficacy (e.g. patients better identify their own problems, make decisions, and take appropriate actions)<br><br>-Increased self-management goal setting<br><br>-Increased patient	-Improved chronic disease outcomes<br><br>-Increased health literacy

Care Coordination Domains		Structure (Inputs)	Process (Activities)	Short Term Outcomes	Long Term Outcomes
			set self-management goals that are recorded in their care plan<br>-Patients engage in peer mentoring/counseling groups to learn from others with similar health conditions	satisfaction	
**Communication**	Interpersonal Communication	- has an effective system to ensure timely and effective communication with patients and outside care providers<br>- provides access to care coordinators who directly contact (e.g. in person, phone, secure message) patients needing care coordination to assist them in carrying out care plans	-Care coordinators communicate with outside care providers to ensure timely information sharing and coordination of care<br>-Care coordinators communicate seamlessly and frequently with patients needing care coordination to assist them in carrying out care plans	-Increased patient satisfaction	-Reduced costs<br>-Improved clinical outcomes<br>-Reduced medical errors<br>-Reduced duplicative testing
	Information Transfer	- provides access to care coordinators to ensure that needed clinical information is transferred between the medical home and outside entities (e.g. hospitals, VNA, specialists)<br>- has needed patient information in the electronic health record system available to all primary care team members	-Care coordinators ensure the timely transfer and receipt of needed patient information (e.g. test results, specialist notes)<br>-Patients experience a seamless transition between outpatient and specialist care with close follow up<br>-All entities providing care for the patient possess all needed care information	-Enhanced information sharing between primary care team and outside providers<br>-Improved patient and staff satisfaction	-Reduced duplicative tests<br>-Reduced number of specialist visits<br>-Reduced adverse drug events<br>-Reduced medical costs
**Patient Assessment and Support**	Needs Assessment	- has a process to routinely and proactively assess needs of patients with complex health issues and/or uncontrolled chronic illness	-Complex patients have a comprehensive assessment of their medical and psychosocial needs by care coordinators<br>-Care coordinators regularly collect and review patient information from multiple sources (e.g. medical record, discussion with care team and patient) to develop a complete assessment of patient's needs	-Increased patient satisfaction<br>-Enhanced patient self-efficacy<br>-Better alignment with community resources	-Improved clinical outcomes
	Monitoring,	- has a system for identifying and	-Care coordinators systematically follow up	-Increased contact	-Reduced ambulatory

Care Coordination Domains		Structure (Inputs)	Process (Activities)	Short Term Outcomes	Long Term Outcomes
	Follow Up, and Responding to Status Changes	responding to changes in status for patients receiving care coordination<br>- provides access to care coordinators who monitor, follow up, and respond to changes in status	on tests, treatments, and services for patients receiving care coordination<br>-Care coordinators closely monitor patients receiving care coordination and respond to changes in a timely fashion<br>-Care coordinators routinely review patient status	between primary care team and patients receiving care coordination	sensitive admissions<br>-Reduced hospital re-admissions<br>-Reduced hospital/ER utilization<br>-Reduced medical errors
	Linkage to Community Resources	- has a comprehensive listing of supportive community resources (e.g. financial, social, educational)<br>- provides access to care coordinators who link patients to community resources	-Care coordinators constantly seek to link patients to all appropriate supportive resources in their community to maximize achievement of all care plan goals	-Increased patient satisfaction<br>-Increased patient access to community based services	-Improved patient outcomes
**Care Transitions**		- receives timely notification of all patients discharged from hospitals and other acute care facilities<br>- provides access to care coordinators who contact patients in a timely fashion after hospital/acute care discharge	-Patients experience a seamless transition between inpatient and outpatient care with close follow up and attention to changes in care plan<br>-Care coordinators receive timely notifications of all hospital discharges<br>-Care coordinators make contact with patients by phone and, if necessary, in person within 48 hours post discharge to assess patient status and support care plan as needed<br>-Care coordinators reconcile medication at every transition event	-Reduced 30 day re-admission rate<br>-Increased patient satisfaction	-Reduced overall medical costs<br>-Reduced mortality<br>-Reduced medication errors
