# Supplementary material for: Development and validation of the Medical Home Care Coordination Survey for assessing care coordination in the primary care setting from the patient and provider perspectives
Source: BMC Health Serv Res. 2015 Jun 7;15:226. doi: 10.1186/s12913-015-0893-1 (PMC4482098; doi:10.1186/s12913-015-0893-1)
Supplement: Supplementary file 2 — Final MHCCS-P. [file 12913_2015_893_MOESM2_ESM.pdf]

Medical Home Care Coordination Survey – Patient (MHCCS-P)

Additional file 2. Final MHCCS-P

SURVEY INSTRUCTIONS

- Do your best to answer each question based on your opinion of the care you received from your primary care provider's office in the **last 12 months**.
- Please **only** select **one answer per question**.
- Please answer all questions by checking the box to the left of your answer.
- Please answer all questions honestly and completely.
- Sometimes you will be told to **skip** over some questions in the survey based on your answer. If this happens, the arrow beside your answer will tell you what question to answer next.

  For example, if you answered "No" below, your next question to answer will be Question 20. You can skip all questions between your current question and Question 20.

  ☐ Yes
  ☒ No → **If No**, Go to **QUESTION 20**

Please read this to better understand the survey questions:

COORDINATING YOUR CARE means making sure everyone (including you, your primary care provider, people who take care of you, and other doctors you see) have all the information needed to take care of your health.

A PRIMARY CARE PROVIDER is a doctor or other health care provider, such as an advanced practice nurse (APRN) or a physician assistant (PA), who you go to for all or most of your routine health care needs.

A PRIMARY CARE TEAM is a group of people in your primary care provider's office who work together to take care of you. This includes your primary care provider, the medical assistant and nurses. Sometimes it may include other health care providers like diabetes educators, nutritionists and pharmacists.

PLEASE BEGIN THE SURVEY

Community Health Center, Inc.

Medical Home Care Coordination Survey – Patient (MHCCS-P)

1. Our records show that you visited your primary care provider's office in the last 12 months. Is this true?

- ☐ Yes
- ☐ No → **If No**, **PLEASE STOP HERE** and return the survey in the enclosed prepaid envelope.

2. Do you have a care plan?
(A care plan is something that you and your primary care provider make together on what needs to be done to take care of your health.)

- ☐ Yes
- ☐ No → **If No**, Go to **QUESTION 6**

3. My primary care team…

a. …asks for my ideas when we make a plan for my care.

- ☐ Never
- ☐ Rarely
- ☐ Sometimes
- ☐ Usually
- ☐ Always
- ☐ I Don't Know

b. …follows through with the care plan it creates with me.

- ☐ Never
- ☐ Rarely
- ☐ Sometimes
- ☐ Usually
- ☐ Always
- ☐ I Don't Know

4. My primary care team helps me plan so I can take care of my health even when things change or when unexpected things happen.

- ☐ Disagree
- ☐ Somewhat Disagree
- ☐ Neither Agree nor Disagree
- ☐ Somewhat Agree
- ☐ Agree
- ☐ I Don't Know

5. Someone on my primary care team helps me set goals for taking care of my health.

- ☐ Never
- ☐ Rarely
- ☐ Sometimes
- ☐ Usually
- ☐ Always
- ☐ I Don't Know

6. Someone on my primary care team…

a. …asks me about what I need for support, like care programs, financial services, equipment and transportation.

- ☐ Disagree
- ☐ Somewhat Disagree
- ☐ Neither Agree nor Disagree
- ☐ Somewhat Agree
- ☐ Agree
- ☐ I Don't Know

b. …gives me information about services offered at their office or in my community, like counseling programs, support groups and rehabilitation programs.

- ☐ Disagree
- ☐ Somewhat Disagree
- ☐ Neither Agree nor Disagree
- ☐ Somewhat Agree
- ☐ Agree
- ☐ I Don't Know

c. …encourages me to attend programs in my community that could help me, like support groups and exercise classes.

- ☐ Disagree
- ☐ Somewhat Disagree
- ☐ Neither Agree nor Disagree
- ☐ Somewhat Agree
- ☐ Agree
- ☐ I Don't Know

7. In the **last 12 months**, have you done any lab tests, like blood tests and x-rays?

- ☐ Yes
- ☐ No → **If No**, Go to **QUESTION 10**

8. I get the results of my lab tests in a timely manner.

- ☐ Never
- ☐ Rarely
- ☐ Sometimes
- ☐ Usually
- ☐ Always
- ☐ I Don't Know

9. Someone on my primary care team…

a. …tells me all my test results, good and bad.

- ☐ Never
- ☐ Rarely
- ☐ Sometimes
- ☐ Usually
- ☐ Always
- ☐ I Don't Know

b. …helps me understand what my lab tests, x-rays or other test results mean.

- ☐ Never
- ☐ Rarely
- ☐ Sometimes
- ☐ Usually
- ☐ Always
- ☐ I Don't Know

10. How long ago were you hospitalized, if at all? *(Hospitalized means you were admitted to a hospital for treatment for a condition. This does not include Emergency Room visits.)*

- ☐ Less than 1 month ago
- ☐ Between 1 and 6 months ago
- ☐ Between 6 and 12 months ago
- ☐ I **was not hospitalized** in the last 12 months → Go to **QUESTION 12**

11. After I leave the hospital, **my primary care team**…

a. …knows about the care I received from the hospital.

- ☐ Disagree
- ☐ Somewhat Disagree
- ☐ Neither Agree nor Disagree
- ☐ Somewhat Agree
- ☐ Agree
- ☐ I Don't Know

b. …helps me get back on my feet after I leave the hospital.

- ☐ Disagree
- ☐ Somewhat Disagree
- ☐ Neither Agree nor Disagree
- ☐ Somewhat Agree
- ☐ Agree
- ☐ I Don't Know

c. …knows about new prescriptions or if there was a change in my medication.

- ☐ Disagree
- ☐ Somewhat Disagree
- ☐ Neither Agree nor Disagree
- ☐ Somewhat Agree
- ☐ Agree
- ☐ I Don't Know

12. In general, the care I receive is well coordinated.

- ☐ Disagree
- ☐ Somewhat Disagree
- ☐ Neither Agree nor Disagree
- ☐ Somewhat Agree
- ☐ Agree
- ☐ I Don't Know

13. In general, how would you **rate your overall health**?

- ☐ Poor
- ☐ Fair
- ☐ Good
- ☐ Very Good
- ☐ Excellent

14. In general, how would you **rate the care you receive** at your primary care provider's office?

- ☐ Poor
- ☐ Fair
- ☐ Good
- ☐ Very Good
- ☐ Excellent

15. What is the highest level of education you have received?

- ☐ No schooling
- ☐ Grade 1 through 12, What Grade: ________
- ☐ High School or GED Completed
- ☐ Some College Completed
- ☐ Associate's Degree Completed
- ☐ Bachelor's Degree Completed
- ☐ Advanced Degree Completed
- ☐ Other, Please Specify: ___________________
