# Supplementary material for: Development and validation of the Medical Home Care Coordination Survey for assessing care coordination in the primary care setting from the patient and provider perspectives
Source: BMC Health Serv Res. 2015 Jun 7;15:226. doi: 10.1186/s12913-015-0893-1 (PMC4482098; doi:10.1186/s12913-015-0893-1)
Supplement: Supplementary file 3 — Final MHCCS-H. [file 12913_2015_893_MOESM3_ESM.pdf]

Additional file 3. Final MHCCS-H

SURVEY INSTRUCTIONS

- Do your best to answer each question based on your opinion of the care coordination provided at your primary care practice/health center in the **last 12 months**.
- Please **only** select **one answer per question** unless specified otherwise.
- Please answer all questions by checking the box to the left of your answer.
- Please answer all questions honestly and completely.

Please read this to better understand the survey questions:

CARE COORDINATION* is the "deliberate organization of patient care activities between two or more participants (including the patient) involved in a patient's care to facilitate the appropriate delivery of health care services. Organizing care involves the marshaling of personnel and other resources needed to carry out all required patient care activities and is often managed by the exchange of information among participants responsible for different aspects of care".

*Ref: McDonald KM, Schultz E, Albin L, Pineda N, Lonhart J, Sundaram V, Smith-Spangler C, Brustrom J, and Malcolm E. Care Coordination Atlas Version 3 (Prepared by Stanford University under subcontract to Battelle on Contract No. 290-04-0020). AHRQ Publication No. 11-0023-EF. Rockville, MD: Agency for Healthcare Research and Quality. November 2010.

PLEASE BEGIN THE SURVEY

1. The primary care team is made up of members with clearly defined roles, such as patient self management education, proactive follow up and resource coordination.

- ☐ Disagree
- ☐ Somewhat Disagree
- ☐ Neither Agree nor Disagree
- ☐ Somewhat Agree
- ☐ Agree
- ☐ I Don't Know

2. The primary care team and patients share responsibilities in managing patients' health.

- ☐ Disagree
- ☐ Somewhat Disagree
- ☐ Neither Agree nor Disagree
- ☐ Somewhat Agree
- ☐ Agree
- ☐ I Don't Know

3. The primary care team is characterized by collaboration and trust.

- ☐ Disagree
- ☐ Somewhat Disagree
- ☐ Neither Agree nor Disagree
- ☐ Somewhat Agree
- ☐ Agree
- ☐ I Don't Know

4. The primary care team works with patients to help them understand their roles and responsibilities in care.

- ☐ Disagree
- ☐ Somewhat Disagree
- ☐ Neither Agree nor Disagree
- ☐ Somewhat Agree
- ☐ Agree
- ☐ I Don't Know

5. The primary care team **uses electronic data to…**

a. …identify patients with complex health needs.

- ☐ Never
- ☐ Rarely
- ☐ Sometimes
- ☐ Usually
- ☐ Always

☐ I Don't Know

b. …monitor and track patient health indicators and outcomes.

- ☐ Never
- ☐ Rarely
- ☐ Sometimes
- ☐ Usually
- ☐ Always

☐ I Don't Know

6. The primary care team **uses an electronic health record system or other electronic systems to…**

a. …support the documentation of patient needs.

- ☐ Never
- ☐ Rarely
- ☐ Sometimes
- ☐ Usually
- ☐ Always

☐ I Don't Know

b. …develop care plans.

- ☐ Never
- ☐ Rarely
- ☐ Sometimes
- ☐ Usually
- ☐ Always

☐ I Don't Know

c. …determine clinical outcomes.

- ☐ Never
- ☐ Rarely
- ☐ Sometimes
- ☐ Usually
- ☐ Always

☐ I Don't Know

7. The primary care team…

a. …informs patients about any diagnosis in a way that they can understand.

- ☐ Disagree
- ☐ Somewhat Disagree
- ☐ Neither Agree nor Disagree
- ☐ Somewhat Agree
- ☐ Agree

☐ I Don't Know

b. …helps patients understand all of the choices for their care.

- ☐ Disagree
- ☐ Somewhat Disagree
- ☐ Neither Agree nor Disagree
- ☐ Somewhat Agree
- ☐ Agree

☐ I Don't Know

c. …considers and respects patients' values, beliefs and traditions when recommending treatments.

- ☐ Disagree
- ☐ Somewhat Disagree
- ☐ Neither Agree nor Disagree
- ☐ Somewhat Agree
- ☐ Agree

☐ I Don't Know

8. The primary care team's care coordination activities are based upon ongoing assessment of patient needs.

- ☐ Disagree
- ☐ Somewhat Disagree
- ☐ Neither Agree nor Disagree
- ☐ Somewhat Agree
- ☐ Agree

☐ I Don't Know

9. The primary care team…

a. …asks for patients' input when making a plan for their care.

- ☐ Never
- ☐ Rarely
- ☐ Sometimes
- ☐ Usually
- ☐ Always

☐ I Don't Know

b. …helps make care plans that patients can follow in their daily life.

- [ ] Never
- [ ] Rarely
- [ ] Sometimes
- [ ] Usually
- [ ] Always
- [ ] I Don't Know

c. …develops care plans that incorporate plans recommended by other health care providers that patients see.

- [ ] Never
- [ ] Rarely
- [ ] Sometimes
- [ ] Usually
- [ ] Always
- [ ] I Don't Know

10. The primary care team reviews and updates patients' care plan with them.

- [ ] Disagree
- [ ] Somewhat Disagree
- [ ] Neither Agree nor Disagree
- [ ] Somewhat Agree
- [ ] Agree
- [ ] I Don't Know

11. The primary care team…

a. …gives patients a copy of their care plan.

- [ ] Never
- [ ] Rarely
- [ ] Sometimes
- [ ] Usually
- [ ] Always
- [ ] I Don't Know

b. …follows through with the care plan.

- [ ] Never
- [ ] Rarely
- [ ] Sometimes
- [ ] Usually
- [ ] Always
- [ ] I Don't Know

c. …uses patients' care plan to follow progress.

- [ ] Never
- [ ] Rarely
- [ ] Sometimes
- [ ] Usually
- [ ] Always
- [ ] I Don't Know

12. The primary care team helps patients plan so they can take care of their health even when things change or when unexpected things happen.

- [ ] Disagree
- [ ] Somewhat Disagree
- [ ] Neither Agree nor Disagree
- [ ] Somewhat Agree
- [ ] Agree
- [ ] I Don't Know

13. Someone on the primary care team…

a. …helps patients set goals for managing their health.

- [ ] Never
- [ ] Rarely
- [ ] Sometimes
- [ ] Usually
- [ ] Always
- [ ] I Don't Know

b. …checks to see if patients are reaching their goals.

- [ ] Never
- [ ] Rarely
- [ ] Sometimes
- [ ] Usually
- [ ] Always
- [ ] I Don't Know

14. The primary care practice/health center…

a. …has behavior change interventions readily available for patients as part of routine care.

- [ ] Disagree
- [ ] Somewhat Disagree
- [ ] Neither Agree nor Disagree
- [ ] Somewhat Agree
- [ ] Agree
- [ ] I Don't Know

b. …has peer support readily available for patients as part of routine care.

- ☐ Disagree
- ☐ Somewhat Disagree
- ☐ Neither Agree nor Disagree
- ☐ Somewhat Agree
- ☐ Agree
- ☐ I Don't Know

15. Someone on the primary care team asks patients about what they need for support, such as care programs, financial services, equipment and transportation.

- ☐ Disagree
- ☐ Somewhat Disagree
- ☐ Neither Agree nor Disagree
- ☐ Somewhat Agree
- ☐ Agree
- ☐ I Don't Know

16. Someone on the primary care team offers patients the opportunity to learn more about managing their health, such as with group appointments, support groups and patient education.

- ☐ Disagree
- ☐ Somewhat Disagree
- ☐ Neither Agree nor Disagree
- ☐ Somewhat Agree
- ☐ Agree
- ☐ I Don't Know

17. Someone on the primary care team…

a. …gives patients information about additional supportive services offered at the practice/health center or in their community, such as counseling programs, support groups or rehabilitation programs.

- ☐ Disagree
- ☐ Somewhat Disagree
- ☐ Neither Agree nor Disagree
- ☐ Somewhat Agree
- ☐ Agree
- ☐ I Don't Know

b. …encourages patients to attend programs in their community that could help them, such as support groups or exercise classes.

- ☐ Disagree
- ☐ Somewhat Disagree
- ☐ Neither Agree nor Disagree
- ☐ Somewhat Agree
- ☐ Agree
- ☐ I Don't Know

c. …connects patients to needed services, such as transportation or home care.

- ☐ Disagree
- ☐ Somewhat Disagree
- ☐ Neither Agree nor Disagree
- ☐ Somewhat Agree
- ☐ Agree
- ☐ I Don't Know

18. When patients are discharged from the hospital, **the primary care team…**

a. …is informed about the care patients received from the hospital.

- ☐ Disagree
- ☐ Somewhat Disagree
- ☐ Neither Agree nor Disagree
- ☐ Somewhat Agree
- ☐ Agree
- ☐ I Don't Know

b. …receives information from the hospital about new prescriptions or if there was a change in medication.

- ☐ Disagree
- ☐ Somewhat Disagree
- ☐ Neither Agree nor Disagree
- ☐ Somewhat Agree
- ☐ Agree
- ☐ I Don't Know

19. When patients are discharged from the hospital, their primary care medical record includes a discharge summary in a timely manner.

- ☐ Never
- ☐ Rarely
- ☐ Sometimes
- ☐ Usually
- ☐ Always
- ☐ I Don't Know

20. When patients are discharged from the hospital and there are test results pending, their primary care medical record includes the test results within 2 weeks.

- ☐ Never
- ☐ Rarely
- ☐ Sometimes
- ☐ Usually
- ☐ Always
- ☐ I Don't Know

21. In general, how would you rate the coordination of care provided at your primary care practice/health center?

- ☐ Poor
- ☐ Fair
- ☐ Good
- ☐ Very Good
- ☐ Excellent

22. What is your principal role in your health center?
*Please select all that apply

- ☐ Administrator
- ☐ Primary Care Physician (e.g. MD, DO)
- ☐ Nurse Practitioner (e.g. APRN)
- ☐ Registered Nurse (e.g. RN)
- ☐ Nurse Care Coordinator
- ☐ Other, please specify: ___________________

23. What is your gender?

- ☐ Male
- ☐ Female
